# Distinguishing Adenocarcinomas from Granulomas in the CT scan of the chest: performance degradation evaluation in the automatic segmentation framework

**DOI:** 10.1186/s13104-021-05502-1

**Published:** 2021-03-09

**Authors:** Mahsa Bank Tavakoli, Mahdi Orooji, Mehdi Teimouri, Ramita Shahabifar

**Affiliations:** 1 Faculty of New Sciences and Technologies, University of Tehran, Tehran, Iran; 2 Department of Electrical and Computer Engineering, Tarbiat Modares University, Tehran, Iran; 3 Kerman University Of Medical Science, Kerman, Iran

**Keywords:** Lung cancer, Computed tomography of the chest, Computer-Aided Diagnosis, Radiomic features, Vessel tortuosity

## Abstract

**Objective:**

The most common histopathologic malignant and benign nodules are Adenocarcinoma and Granuloma, respectively, which have different standards of care. In this paper, we propose an automatic framework for the diagnosis of the Adenocarcinomas and the Granulomas in the CT scans of the chest from a private dataset. We use the radiomic features of the nodules and the attached vessel tortuosity for the diagnosis. The private dataset includes 22 CTs for each nodule type, i.e., adenocarcinoma and granuloma. The dataset contains the CTs of the non-smoker patients who are between 30 and 60 years old. To automatically segment the delineated nodule area and the attached vessels area, we apply a morphological-based approach. For distinguishing the malignancy of the segmented nodule, two texture features of the nodule, the curvature Mean and the number of the attached vessels are extracted.

**Results:**

We compare our framework with the state-of-the-art feature selection methods for differentiating Adenocarcinomas from Granulomas. These methods employ only the shape features of the nodule, the texture features of the nodule, or the torsion features of the attached vessels along with the radiomic features of the nodule. The accuracy of our framework is improved by considering the four selected features.

**Supplementary Information:**

The online version contains supplementary material available at 10.1186/s13104-021-05502-1.

## Introduction

Diagnosis of malignant nodules in early stages via Computed Tomography (CT) scans is an important step for reducing lung cancer mortality [1]. In this regard, Computer Aided Diagnosis (CADx) systems are presented that use radiomic features of suspicious nodules in CT images [2]. The most common histopathologic malignant nodules that appear as subsolid in CT images are adenocarcinomas; as a result, characterizing adenocarcinomas in CT images is challenging [3]. Also, granulomas are a broad group of benign nodules that appear similar in size, shape, and appearance to lung cancers on CT [4]. Since in most imaging-based evaluations, the benign nodules (such as granulomas) usually look similar to the cancerous nodules (such as adenocarcinomas), a biopsy is often necessary for a certain diagnosis of the cancerous ones. Unfortunately, the biopsy of the lung is an invasive, painful, and costly procedure.

In this paper, we introduce an automatic CADx that uses the radiomic features of the nodules plus the tortuosity and the number of the vessels attached to the nodules for distinguishing granulomas from adenocarcinomas in the lung CTs of the private dataset. In our system, first, the nodules and the attached vessels are segmented. For segmenting the delineated nodules and the attached vessels, we apply the morphological framework introduced in [5]. The radiomics features of the nodules plus the tortuosity and the number of the vessels attached to the nodules are then extracted. The tortuosity features are the curvature Mean, the fractional dimension Mean, and the distance metric Mean of the attached vessels. The radiomic features are 855 features, including the shape, the sharpness, and the texture features of the nodules. Next, a subset of four features is selected by the forward selection algorithm. These features are the standard deviation of the correlation feature and the kurtosis of the diagonal gradient images from the nodule area and the curvature Mean and the number of the attached vessels. The selected features are extracted from the manual and automatic annotations of the nodules and the attached vessels in the CT images. Finally, the Support Vector Machine (SVM) classifications with threefold cross-validation are performed on the selected features. Our CADx system, such as other CADx systems, is introduced to help physicians for diagnosing cancerous nodules (Adenocarcinoma) from benign confounders (Granuloma). This system has a significant impact on the treatment road by increasing the accuracy of the diagnosis and reducing the necessity of repeated biopsy.

## Main text

### Data

We employ a database from the Afzalipour Hospital of Kerman. The database includes the CTs of 44 non-smoker patients, who were between 30 and 60 years old. Each case had a dubious nodule of size $11.91\pm 4.36$mm. The database consists of 22 CTs for each nodule type, i.e., Adenocarcinoma and Granuloma. Smoking is the main cause of emphysema signs [6]. Emphysema can also be diagnosed by low attenuation area in lung CT images [7]. The framework in [5] (for the automatic segmentation of nodules) applies a threshold-based Region Growing (RG) algorithm. As a result, the boundaries of the segmented nodules are not accurate. So, we do not consider the CTs of smoking people. All the CT scans were collected as part of an Institutional Review Board-approved, HIPAA-compliant protocol. In addition, these CTs were constructed by the Siemens scanner machine with exposure 120 KVp, slice thickness of 1–5 mm, and an X-ray tube current of 41–200 mAs. All the CTs have 100 to 400 slices. The resolution of each slice is $512\times 512$ pixels. The type of each nodule is also described using the microscopic analysis of the nodule specimen gathered by biopsy and/or surgical resection. Moreover, the CTs consist of three popular types of nodules (containing solid, part-solid, and non-solid). The regions of the nodule and the connected vessels in the database are also annotated by a skilled radiologist.

### Methods

We employ the framework introduced in [5] for the segmentation of each nodule and the connected vessels in the Region Of Interest (ROI) (i.e, a volume around the seed point).

**Fig. 1:**
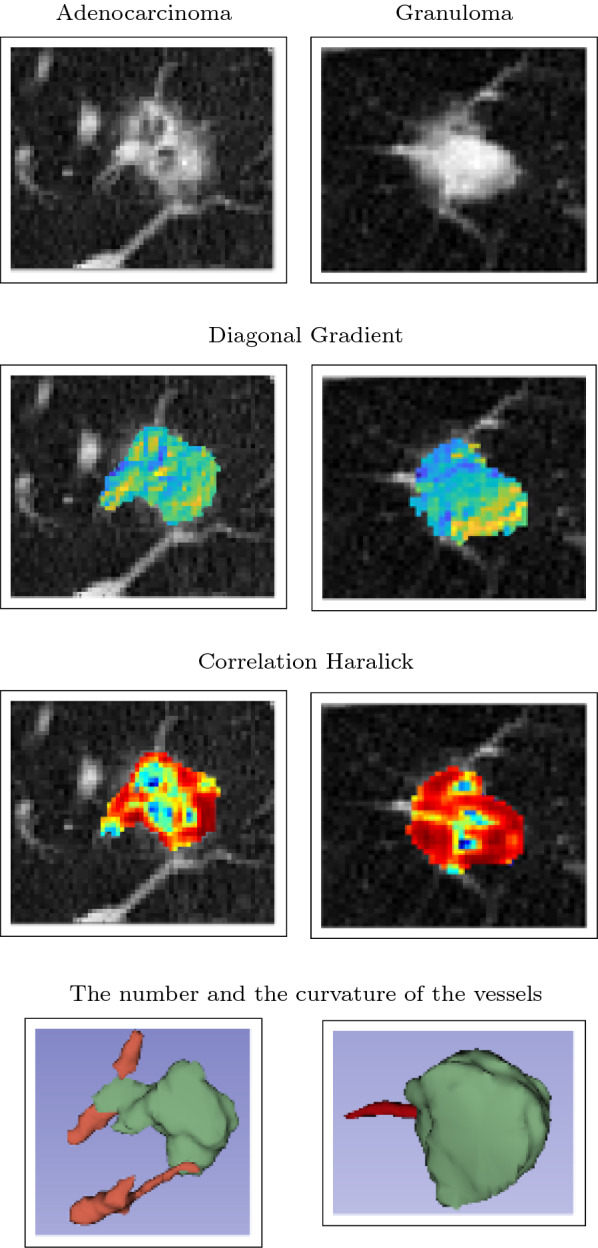
The illustration of the 4 selected features for a granuloma and an adenocarcinoma

Then, three sets of 3D features, including 830 texture features, 13 shape features, and 12 sharpness features, are automatically extracted for nodule characterization. We also extract 4 features, including 3 tortuosity features and the number of the attached vessels, from the segmented vessels area. We mentioned the details about the features of the nodule and the attached vessels in the Additional file 1. Hence, a feature set including 859 features is extracted for each CT images in the dataset. After that, a subset of four features is selected by the forward selection algorithm. The selected features are the standard deviation of the correlation feature and the kurtosis of the diagonal gradient images from the nodule area, as well as the curvature Mean and the number of the attached vessels from the vessels area. Figure 1 illustrates the selected features for a granuloma and an adenocarcinoma in the dataset.

We employ a SVM classifier in conjunction with four selected features to discriminate between adenocarcinomas and granulomas. The classifier employs the linear kernel. Moreover, we use threefold cross-validation for obtaining classification results. The performance of the classifiers is measured via the Area Under ROC Curve (AUC) of the (ROC) curve.

To compare the classification accuracy of the proposed feature selection method to the state-of-the-art feature selection methods [4, 8, 9], we introduce eight different scenarios that are performed by eight different SVM classifiers. The scenarios correspond to the combination of two different annotations and four different feature sets. Two types of annotation are obtained by two segmentation approaches, manual and the automatic framework. The manual annotations are made by the radiologist. The automatic annotations are resulted by the introduced framework in [5].

We consider the feature sets as follows. In two scenarios, four selected features ($FS_{nv}$) are employed. The feature set $FS_{nv}$ includes the standard deviation of the correlation feature and the kurtosis of the diagonal gradient images from the nodule area, as well as the curvature Mean and the number of the attached vessels. In the other two scenarios, we consider two texture features of the nodule ($FS_n$). The feature set $FS_n$ includes the standard deviation of the correlation feature and the kurtosis of the diagonal gradient images. These texture features are also used in [9] for distinguishing between adenocarcinomas and granulomas.

In two other scenarios, three shape features of the nodule area proposed by [4] ($FS_s$) are used in conjunction with two annotations. The feature set $FS_s$ includes roughness, convexity, and sphericity. Also, to compare the classification accuracy of the proposed feature selection method to the state-of-the-art feature selection method [8], two more scenarios are considered. In [8], 12 tortuosity features of the vessels attached to nodules are introduced for distinguishing granulomas from adenocarcinomas. We select two features from these 12 features by the forward selection algorithm. Two selected features ($FS_v$) are the Max value of the maximum curvature values of the vessel branches and the 4th bin value from the Histogram of torsion measurements of the branches. These features are important for distinguishing benign nodules from the malignant ones because the malignant one tends to pull the vessels toward itself for better feeding and growing. As a result, the vessels which reach the malignant nodule are more torsion. Hence, in these scenarios, two selected tortuosity features of the attached vessels $FS_v$ are used in conjunction with two annotations.

The classification results of manually and automatically segmented nodules are provided in Table 1. The classification results of the segmented nodules using the feature set (proposed in this paper) $FS_{nv}$, three shape features (introduced in [4]) $FS_s$, two selected texture features (introduced in [9]) $FS_n$, and two selected tortuosity features of the attached vessels (used in [8]) $FS_v$ are also reported in Table 1. As it can be seen in Table 1, the AUC values of the classifications by the feature set $FS_{nv}$ improve compared to those of the feature sets $FS_n$, $FS_s$, and $FS_v$ for both manual and automatically segmented nodules.

**Table 1:** The classification results of manually and automatically segmented nodules

Annotation	Feature Set	AUC
Manual	4 Selected Features ($FS_{nv}$)	0.8874
Manual	2 Selected Texture Features ($FS_n$) by [9]	0.8595
Manual	2 Selected tortuosity Features by [8] ($FS_v$)	0.7333
Manual	3 Selected Shape Features by [4] ($FS_s$)	0.6342
The Automatic Framework [5]	$FS_{nv}$	0.7583
The Automatic Framework [5]	$FS_n$	0.7211
The Automatic Framework [5]	$FS_v$	0.6756
The Automatic Framework [5]	$FS_s$	0.5444

### Discussion

In this study, we investigated the role of automatic segmentation of the delineated pulmonary nodule and the attached vessels, as well as computerized image analysis to identify a set of nodule texture features and the attached vessels tortuosity that best distinguish adenocarcinomas from granulomas on the CT scans of the chest (from the private dataset). Our study revealed that the standard deviation of the correlation feature and the kurtosis of the diagonal gradient images from the nodule area, as well as the curvature Mean and the number of the attached vessels from the vessels area, were the most predictive and discriminating features. The performance of the SVM classifier has resulted in an AUC of 75.83% and 88.74%, on the texture and tortuosity features $FS_{nv}$, using the automatic framework (proposed in [5]) and the manual segments (labeled by an expert radiologist), respectively. The performance of the SVM classifier using the manual segments is about $13\%$ better than that of the automatic segments. However, the time needed for the annotation of each nodule and the attached vessels by a radiologist is much higher than that of the automatic segmentation. Hence, in the process of lung cancer screening in which the number of cases is high, using the automatic segmentation would be much more cost-effective.

In [9], 12 texture features of the nodule are employed to discriminate granulomas from adenocarcinomas. We selected two features ($FS_n$) from these texture features by the forward selection algorithm. We then extracted two selected texture features $FS_n$ from our dataset to differentiate the granulomas from the adenocarcinomas. In [4], three shape features of the nodule ($FS_s$), including roughness, convexity, and sphericity are also employed, to discriminate granulomas from adenocarcinomas. In our study, we also extracted these shape features from our dataset to differentiate the granulomas from the adenocarcinomas. Moreover, In [8], 12 tortuosity features of the attached vessels are employed to discriminate granulomas from adenocarcinomas. We selected two features ($FS_v$) from these 12 tortuosity features of the attached vessels by the forward selection algorithm. We then extracted two selected tortuosity features $FS_v$ from our dataset.

In the case of applying the segmentation framework in [5], the use of the texture and tortuosity features ($FS_{nv}$) amplified the performance of the SVM classifier compared to those of two selected tortuosity features ($FS_v$), two selected texture features ($FS_n$), or the shape features ($FS_s$) alone, with an increase of 8.27%, 3.72%, and 21.39% in the AUC values, respectively. In the case of using manual segments (labeled by the radiologist), an AUC value of 85.95%, 73.33%, and 63.42% was obtained on two selected texture features ($FS_n$), two selected tortuosity features ($FS_v$), and the shape features ($FS_s$) alone, respectively. As a result, the AUC value of the classifier with the texture and tortuosity features ($FS_{nv}$) improves, in comparison to those of two texture features ($FS_n$), two tortuosity features ($FS_v$), and the shape features ($FS_s$) alone, with a rise of $2.79\%$, $15.41\%$, and $25.32\%$, respectively. This result demonstrates the superiority of the proposed feature selection method compared to the state-of-the-art feature selection methods [4, 8, 9].

## Limitations

Our study did have its limitations which included using datasets consist of one specific type of benign and malignant pathology, i.e. granulomas and adenocarcinomas from only one institution. For a more general conclusion, the introduced frameworks must be evaluated on the independent cohort. As future work, it is valuable to evaluate the discriminability of the features and the classifier in distinguishing other benign conditions such as hamartoma and fibrosis from other types of non-small cell lung cancers like squamous cell carcinomas.

## Supplementary Information

**Additional file 1.**

## Data Availability

The data that support the findings of this study are available from the Kerman University Of Medical Science but restrictions apply to the availability of these data, which were used under license for the current study, and so are not publicly available. Data are however available from the authors upon reasonable request and with permission of the Kerman University Of Medical Science.

## References

[1] Midthun DE (2016). Early detection of lung cancer. F1000Res.

[2] Tharcis P, Kezi Selva Vijila C. Computer-aided diagnosis of lung cancer in computed tomography scans: A review. 2018;14(3):374–88. http://www.eurekaselect.com/node/149175/article

[3] Ko JP, Suh J, Ibidapo O, Escalon JG, Li J, Pass H, Naidich DP, Crawford B, Tsai EB, Koo CW, Mikheev A, Rusinek H (2016). Lung adenocarcinoma: Correlation of quantitative ct findings with pathologic findings. Radiology..

[4] Alilou M, Beig N, Orooji M, Rajiah P, Velcheti V, Rakshit S, Reddy N, Yang M, Jacono F, Gilkeson RC, Linden P, Madabhushi A (2017). An integrated segmentation and shape-based classification scheme for distinguishing adenocarcinomas from granulomas on lung ct. Med Phys.

[5] Tavakoli M, Orooji M, Teimouri M, Shahabifar R (2020). Segmentation of the pulmonary nodule and the attached vessels in the ct scan of the chest using morphological features and topological skeleton of the nodule. IET Image Processing..

[6] Yasunaga K, Chérot-Kornobis N, Edmé JL, Sobaszek A, Boulenguez C, Duhamel A, Faivre JB, Remy J, Remy-Jardin M (2013). Emphysema in asymptomatic smokers: Quantitative ct evaluation in correlation with pulmonary function tests. Diagn Intervent Imaging.

[7] Tanabe N, Muro S, Sato S, Oguma T, Sato A, Hirai T (2018). Fractal analysis of low attenuation clusters on computed tomography in chronic obstructive pulmonary disease. BMC Pulmonary Med.

[8] Alilou M, Orooji M, Beig N, Prasanna P, Rajiah P, Donatelli C, Velcheti V, Rakshit S, Yang M, Jacono F, Gilkeson R, Linden P, Madabhushi A (2018). Quantitative vessel tortuosity: A potential ct imaging biomarker for distinguishing lung granulomas from adenocarcinomas. Sci Rep.

[9] Beig N, Khorrami H, Alilou M, Prasanna P, Braman N, Orooji M, Rakshit S, Bera K, Rajiah P, Ginsberg J, Donatelli C, Thawani R, Yang M, Jacono F, Tiwari P, Velcheti V, Gilkeson R, Linden P, Madabhushi A (2018). Perinodular and intranodular radiomic features on lung ct images distinguish adenocarcinomas from granulomas. Radiology.

