# Supplementary material for: Distinguishing Adenocarcinomas from Granulomas in the CT scan of the chest: performance degradation evaluation in the automatic segmentation framework
Source: BMC Res Notes. 2021 Mar 9;14:87. doi: 10.1186/s13104-021-05502-1 (PMC7942003; doi:10.1186/s13104-021-05502-1)
Supplement: Supplementary file 1 — Additional file 1. [file 13104_2021_5502_MOESM1_ESM.pdf]

Feature Extraction

Three sets of 3D features, including 830 texture features, 13 shape features, and 12 sharpness features, are automatically extracted for nodule characterization. We also extract 4 features, including 3 tortuosity features and the number of the attached vessels, from the segmented vessels area.

Features of the Nodule

The texture features are divided into 3 classes. These classes are the first-order statistics, the second-order statistics, and the features of the texture images. First-order statistics characterize the distribution of voxel intensities within the image region defined by the mask [1]. We use the 6 first-order statistics features, including energy, mean, standard deviation, skewness, kurtosis, and uniformity of the nodule segment. The second-order statistics features which are extracted from the texture matrices are mentioned in Table 1. We also calculate 130 texture images from each nodule segment, which are classified into 8 classes. Then, the 6 first-order features are extracted from each texture image. The texture images are listed in Table 2.

We also extract the shape features of the segmented nodule. The shape features are volume, surface area, surface area to volume ratio, sphericity, compactness 1, compactness 2, spherical disproportion, Feret diameter, major axis length, minor axis length, least axis length, elongation, and flatness [1].

Furthermore, we employ the sharpness features of the nodule boundaries for differentiating the benign nodules from the malignant nodules. This is because the boundaries of the malignant nodules appear more spiculated and lobulated in Computed Tomography (CT) images; however, the boundaries of the benign nodules are smoother [2]. The sharpness features are calculated by the intensities of the voxels on the nodule margin. These features are the difference of the max and min values, the sum of values, sum of squares, sum of logs, arithmetic mean, geometric mean, population variance, sample variance, standard deviation, kurtosis measure, skewness measure, and Second Central Moment (SCM) [3].

Features of the Attached Vessels

The vessel features, including 3 tortuosity features and the number of the attached vessels, are extracted from the segmented vessel area. The tortuosity features are the curvature Mean, the fractal dimension Mean, and the distance metric Mean.

The vascular distance metric is the ratio of the actual vessel path length to the linear distance between its endpoints [4]. All segments of the vessels attached to the nodule in the box around the seed point are first identified. A vessel segment is defined as the path between either two branching points or between a branching point and an endpoint. For each segment, the 3D length is divided by the Euclidean distance between the endpoints. The mean value of the distance metric from all vessel segments is the distance metric Mean.

The fractal dimension is computed for each attached vessel by applying a 3D extension of the well-validated box-counting method [5]. In box-counting, the image volume which encloses the attached vessel is divided into a grid of equal cubes, and the

Table 1 The second-order features which are extracted from the texture matrices.

texture matrix	features
Gray Level Run Length Matrix (GLRLM)	Short Run Emphasis (SRE), Long Run Emphasis (LRE), Gray-Level Nonuniformity (GLN), Run-Length Nonuniformity (RLN), Run Percentage (RP), Low Gray-Level Run Emphasis (LGRE), High Gray-Level Run Emphasis (HGRE), Short Run Low Gray-Level Emphasis (SRLGE), Short Run High Gray-Level Emphasis (SRHGE), Long Run Low Gray-Level Emphasis (LRLGE), Long Run High Gray-Level Emphasis (LRHGE), Gray-Level Variance (GLV), and Run-Length Variance (RLV)
Gray Level Size Zone Matrix (GLSZM)	Small Zone Emphasis (SZE), Large Zone Emphasis (LZE), Gray-Level Nonuniformity (GLN), Zone-Size Nonuniformity (ZSN), Zone Percentage (ZP), Low Gray-Level Zone Emphasis (LGZE), High Gray-Level Zone Emphasis (HGZE), Small Zone Low Gray-Level Emphasis (SZLGE), Small Zone High Gray-Level Emphasis (SZHGE), Large Zone Low Gray-Level Emphasis (LZLGE), Large Zone High Gray-Level Emphasis (LZHGE), Gray-Level Variance (GLV), and Zone-Size Variance (ZSV)
Neigboring Gray Tone Difference Matrix (NGTDM)	coarseness, contrast, busyness, complexity, and strength
Gray Level Dependence Matrix (GLDM)	Small Dependence Emphasis (SDE), Large Dependence Emphasis (LDE), Gray Level Non-Uniformity (GLN), Gray Level Non-Uniformity Normalized (GLNN), Dependence Non-Uniformity (DN), Dependence Non-Uniformity Normalized (DNN), Gray Level Variance (GLV), Dependence Variance (DV), Dependence Entropy (DE), High Gray Level Emphasis (HGLE), Small Dependence Low Gray Level Emphasis (SDLGLE), Large Dependence High Gray Level Emphasis (LDHGLE), and Large Dependence Low Gray Level Emphasis (LDLGLE)

number of cubes containing part of the attached vessel centerline is counted. This process is repeated for different cube sizes. The number of cubes containing the centerline is plotted against the cube size in a double logarithmic plot. The fractal dimension is equivalent to the slope of the fitted line. The fractal dimension mean is the average of the fractal dimensions from the attached vessels.

If a curve is defined in polar coordinates by the radius r as a function of the polar angle θ, then its curvature is defined in Eq. (1),

$$\kappa(\theta) = \frac{\|r^2 + 2\left(\frac{\mathrm{d}r}{\mathrm{d}\theta}\right)^2 - r\frac{\mathrm{d}^2 r}{\mathrm{d}\theta^2}\|}{\left(r^2 + \left(\frac{\mathrm{d}r}{\mathrm{d}\theta}\right)^2\right)^{\frac{3}{2}}}, \tag{1}$$

where $\frac{\mathrm{d}r}{\mathrm{d}\theta}$ and $\frac{\mathrm{d}^2 r}{\mathrm{d}\theta^2}$ are the first and second derivatives of r with respect to θ [6]. We extract the centerlines of the segments in the attached vessels. Then, the curvature values are calculated for all the centerlines points. The curvature mean is the average of all the curvature values.

References

1. S.T. Namin, H.A. Moghaddam, R. Jafari, M. Esmaeil-Zadeh, M. Gity, in *2010 IEEE International Conference on Systems, Man and Cybernetics* (2010), pp. 3774–3779. DOI 10.1109/ICSMC.2010.5641820
2. A. Snoeckx, P. Reyntiens, D. Desbuquoit, M.J. Spinhoven, P.E. Van Schil, J.P. van Meerbeeck, P.M. Parizel, Insights Imaging **9**(1), 73 (2018). DOI 10.1007/s13244-017-0581-2
3. J. Ferreira, J. R., M.C. Oliveira, P.M. de Azevedo-Marques, J Digit Imaging **31**(4), 451 (2018). DOI 10.1007/s10278-017-0029-8

Table 2 The texture images which are extracted from the nodule area.

texture image	imaged features
Haralick	entropy, energy, inertia, inverse difference moment, correlation, information measure of correlation I, information measure of correlation II, sum average, sum variance, sum entropy, difference average, difference variance, difference entropy
Local Binary Pattern (LBP)	one feature with a 3×3 kernel
gray filters	mean, median, standard deviation, and range
gradient filters	x Sobel Edge, y Sobel Edge, diagonal xy Sobel Edge, diagonal yx Sobel Edge, directional gradient, magnitude gradient, sum square of the directional and the magnitude gradients, Kirsch 1, Kirsch 2, Kirsch 3, dx, dy, and diagonal derivative
Laws filters	25 features by 25 5×5 kernels, such as: $L5L5$, $L5E5$, $L5W5$, etc. L, E, and W are one-dimensional filters respectively for Level detection, Edge detection, and Wave detection.
Laws laplace filters	25 features by 25 5×5 kernels, such as: $L5L5$, $L5E5$, $L5W5$, etc.
autocorrelation	one feature
Gabor filters	48 features by a 48th Gabor filter bank

4. M. Helmberger, M. Pienn, M. Urschler, P. Kullnig, R. Stollberger, G. Kovacs, A. Olschewski, H. Olschewski, Z. Bálint, PLOS ONE **9**(1), 1 (2014). DOI 10.1371/journal.pone.0087515. URL `https://doi.org/10.1371/journal.pone.0087515`
5. M. Ge, Q. Lin, W. Lu, in *2006 IEEE International Symposium on Geoscience and Remote Sensing* (2006), pp. 1423–1426. DOI 10.1109/IGARSS.2006.367
6. A. Riccardi, T. Petkov, G. Ferri, M. Masotti, R. Campanini, Med Phys **38**(4), 1962 (2011)
